# Case series: rituximab in the treatment of refractory sight-threatening scleritis

**DOI:** 10.1186/1479-5876-9-S2-P12

**Published:** 2011-11-23

**Authors:** Kiki van Bilsen, P Martin van Hagen, Tom Missotten, Seerp G Baarsma, Robert W Kuijpers, Jan AM van Laar

**Affiliations:** 1 Dept. of Internal Medicine/Clinical Immunology, Erasmus University Medical Center, Rotterdam, The Netherlands; 2 Dept of Opthalmology, Erasmus University Medical Center, Rotterdam, The Netherlands; 3 The Rotterdam Eye Hospital, Rotterdam, The Netherlands

## Purpose

Scleritis is a chronic vasculitis of scleral vessels leading to a substantial amount of morbidity and even blindness. Oral steroids and intensive immunosuppressive treatments are often used to to achieve long-term control of disease. But those drugs have severe adverse effects and there is a significant number of non-responders. We describe six patients with refractory scleritis who received rituximab.

## Methods

A case series of six patients (aged 39-66) with refractory scleritis (4 idiopathic, 2 relapsing polychondritis), including B cell monitoring. One treatment cycle consisted of 1000 mg initially and 1000 mg two week later. Prior to infusion 100 mg methyprednisolon was administered i.v.

## Results

**Figure 1:**
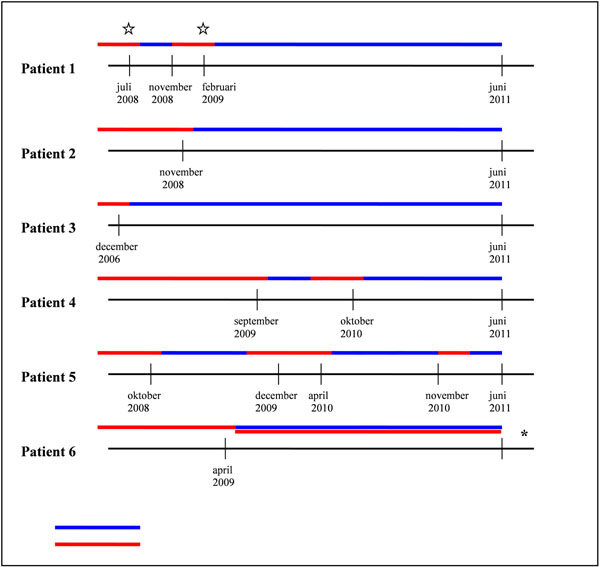


In all patients disease activity decreased. However, in four patients symptoms returned resp. 2,4,6 and 9 months after therapy. In two cases complete remission was achieved. Two patients received a second rituximab cycle successfully. No correlation was found between B cells reoccurrence and refractory disease. [Figure 1]

## Conclusions

Clinical activity of scleritis in all patients decreased after treatment. In two cases scleritis went in complete remission after one cycle of treatment. Rituximab may be a new promising therapeutic tool in the treatment of refractory scleritis. There is no correlation between relapses and B cell recovery.

